# Safety and efficacy of methylenedioxymethamphetamine (MDMA)-assisted psychotherapy in post-traumatic stress disorder: An overview of systematic reviews and meta-analyses

**DOI:** 10.1177/00048674251315642

**Published:** 2025-02-20

**Authors:** Alene Sze Jing Yong, Suzie Bratuskins, Musa Samir Sultani, Brooke Blakeley, Christopher G Davey, J Simon Bell

**Affiliations:** 1 Centre for Medicine Use and Safety, Faculty of Pharmacy and Pharmaceutical Sciences, Monash University, Parkville, VIC, Australia; 2 Neuromedicines Discovery Centre, Monash Institute of Pharmaceutical Sciences, Monash University, Parkville, VIC, Australia; 3 School of Pharmacy, University of Nottingham, Nottingham, UK; 4 Department of Psychiatry, The University of Melbourne, Parkville, VIC, Australia

**Keywords:** Psychedelics, umbrella review, MDMA-assisted therapy, post-traumatic stress disorder

## Abstract

**Objective:**

To critically evaluate published and unpublished systematic reviews and meta-analyses on the safety and efficacy of methylenedioxymethamphetamine-assisted psychotherapy for post-traumatic stress disorder.

**Methods:**

Six bibliometric databases and grey literature were searched from inception to 9 May 2024 for systematic reviews on the safety and efficacy of methylenedioxymethamphetamine (MDMA)-assisted psychotherapy compared to psychotherapy alone among adults with post-traumatic stress disorder. Quality assessment using the AMSTAR-2 tool was conducted independently by two investigators.

**Results:**

Fourteen systematic reviews comprising 20 primary studies involving up to 353 participants were included. All reviews included studies of one-to-three sessions of 50–125 mg MDMA-assisted psychotherapy (some with supplemental dosage) compared to either 25–40 mg of MDMA or inactive placebo with psychotherapy. Four were deemed high quality. Meta-analyses reported substantial benefits of MDMA-assisted psychotherapy in improving post-traumatic stress disorder symptoms (standardised mean difference, 0.8–1.3), response rate (relative risk, 1.3–3.5) and remission rate (relative risk, 2.3–2.9) compared to psychotherapy alone. However, for reviews that assessed the certainty of evidence, the evidence was rated as low to very low certainty due to high risk of bias, indirectness and imprecision. There was moderate-quality evidence that MDMA-assisted psychotherapy was associated with an increased odd of transient adverse events. However, reviews noted reliance on spontaneous rather than systematic adverse event reporting, discrepancies between adverse events reported in published studies and clinical trial registries, and a lack of long-term safety data.

**Conclusion:**

Four high-quality systematic reviews suggest low to very low certainty evidence for efficacy outcomes and moderate to very low quality evidence for safety outcomes.

## Introduction

Post-traumatic stress disorder (PTSD) is a debilitating mental health condition that affects an estimated 3.9% of the global population (Koenen et al., 2017), with a prevalence of up to 27% in countries affected by war (Hoppen et al., 2021). Among the Australian Defence Force members, the 12-month prevalence of PTSD among current serving members was 8%, rising to 18% in those who have transitioned from full-time service (Van Hooff et al., 2018). It is a major public health concern due to its impact on daily functioning (Jellestad et al., 2021) and association with suicidal ideation (Krysinska and Lester, 2010), chronic disease (Edmondson et al., 2013; Roberts et al., 2015; Sumner et al., 2016) and premature death (Boscarino, 2006). There are unique challenges to PTSD treatment, such as low treatment-seeking behaviour and adherence, lack of standardised benchmarks for diagnosis and assessing treatment outcomes, and limited access to high-quality evidence-based trauma-focused treatments (Bryant, 2019; Forbes et al., 2019; Kazlauskas, 2017). Clinical practice guidelines recommended cognitive processing therapy or trauma-focused cognitive behavioural therapy as the first-line management of PTSD (Martin et al., 2021). Even though meta-analyses demonstrate the efficacy of these therapies (Karatzias et al., 2019; Mavranezouli et al., 2020; Watts et al., 2013), it has been estimated that only 54% of those who complete treatment achieve clinically significant improvement (Bradley et al., 2005). The fact that almost half of the population do not improve with current guideline recommended approaches underscores the need for novel and innovative therapeutic interventions.

3,4-Methylenedioxymethamphetamine (MDMA) has been proposed as a potential pharmacological adjunct to psychotherapy after showing promising results in Phase 2 and 3 clinical trials (Mitchell et al., 2021, 2023). Following the Breakthrough Therapy Designation for MDMA-assisted therapy by the US Food and Drug Administration (FDA) in 2017, Lykos Therapeutics filed a new drug application for MDMA used in combination with psychological intervention for adults with PTSD, which was granted a Priority Review by FDA on 9 February 2024 (Lykos Therapeutics, 2024a). However, the application was rejected as of 9 August 2024, with FDA requesting for an additional Phase 3 trial to further investigate MDMA’s safety and efficacy (Lykos Therapeutics, 2024b). While the medical use of psychedelics remains illegal in almost all countries, Australia became the first country to reclassify MDMA and psilocybin from Prohibited Substances (Schedule 9) to Controlled Substances (Schedule 8) in July 2023 (Therapeutic Goods Administration, 2023b). This has allowed authorised prescribers to administer MDMA and psilocybin for the treatment of PTSD and treatment-resistant depression, respectively. The clinical use of psychedelic-assisted psychotherapy has been debated among healthcare professionals, researchers and consumers (The Lancet Regional Health–Europe, 2023). While psychedelic-assisted psychotherapy has the potential to become a novel treatment option for various mental health conditions, concerns have been reported about clinical trial designs and patient expectation in light of extensive media coverage (Muthukumaraswamy et al., 2021; Smith and Appelbaum, 2022; van Elk and Fried, 2023).

There are an increasing number of systematic reviews on MDMA for PTSD. However, there are methodological variations in how these systematic reviews have categorised dosage, types of therapy accompanying MDMA administration, clinical outcomes and populations included (Wheeler and Dyer, 2020). For instance, some reviews stratified the efficacy of MDMA-assisted psychotherapy (MDMA-AP) according to the dosage and types of placebo used (Illingworth et al., 2021; Kisely et al., 2023; Mackey et al., 2022), while the others did not (Bahji et al., 2020, 2023; Hoskins et al., 2021; Tedesco et al., 2021). Most reviews have provided scant information on the psychotherapy sessions. The objective of this overview was to critically evaluate published and unpublished systematic reviews and meta-analyses on the safety and efficacy of MDMA-AP for PTSD. This overview was conducted as part of the evidence review process of developing the Clinical Practice Guideline for the Appropriate Use of MDMA-Assisted Psychotherapy for PTSD.

## Methods

This overview was conducted according to the principles of Overview of Reviews in the Cochrane Handbook for Systematic Reviews of Interventions (Pollock et al., 2020) and reported according to the Preferred Reporting Items for Overviews of Reviews (PRIOR) statement (Gates et al., 2022). The protocol of the overview is available on Open Science Framework (https://osf.io/wjgsm).

### Data sources and search strategy

Six bibliometric databases (MEDLINE, Embase, Allied and Complementary Medicine [AMED], CINAHL, PsycINFO and Cochrane Database of Systematic Reviews) were searched from inception to 9 May 2024 without language restriction. Bibliographic database searches were supplemented by citation searching of included reviews. A grey literature search was conducted on 9 May 2024 using the same keywords via Google to identify government reports, conference proceedings or unpublished reports. The first 10 pages of the search results were screened.

The search strategy was developed using a combination of Medical Subject Headings (MeSH) and specific keywords. The keywords related to ‘MDMA’ (such as N-Methyl-3,4-methylenedioxyamphetamine, methamphetamine, ecstasy, MDMA assisted psychotherapy, psychedelic assisted psychotherapy, substance assisted psychotherapy) AND PTSD (such as post-traumatic stress disorder, stress disorders) AND ‘systematic review or meta-analysis’. The detailed search strategy for each database is provided in Supplementary Table S1.

### Selection criteria

After removing duplicates, the titles and abstracts of all search results were screened independently by two reviewers (A.S.J.Y. and M.S.S.) using Covidence (Veritas Health Innovation, n.d.). Full texts of articles meeting the inclusion and exclusion criteria were obtained and screened against pre-defined eligibility criteria. The list of articles for possible inclusion was compared between the reviewers, and any disagreements were resolved by discussion or involvement of a third reviewer when necessary.

Reviews were included based on the following criteria: (1) Population: adults (18 years or older) with PTSD, (2) Intervention: MDMA-AP, (3) Comparison: psychotherapy with active or inactive placebo, (4) Outcome: benefits (measured as improvements in PTSD symptoms, remission or others) or risks (measured as adverse events) of intervention, (5) Study design: systematic review with or without meta-analysis of randomised controlled trials (RCTs) or observational studies. For the purpose of this overview, a systematic review was defined as a review that conducted a comprehensive, documented and reproducible database search with quality assessment of primary studies.

### Data extraction

Information from the reviews, such as last search date, study design, number of studies and participants, population, intervention, comparator, outcomes, and methods for assessing methodology quality and certainty of evidence, were extracted by A.S.J.Y. and checked by B.B. In cases where reviews encompassed a broader range of interventions or outcomes, only data relevant to the MDMA for PTSD were extracted.

### Overlapping reviews

The degree of overlap in primary studies included in each of the systematic reviews was estimated using the corrected covered area (CCA) method proposed by Pieper et al. (2014). A CCA score of 0–5% denoted a ‘slight overlap’, 6–10% denoted a ‘moderate overlap’, 11–15% denoted a ‘high overlap’ and anything exceeding 15% were classified as ‘very high overlap’ (Pieper et al., 2014).

### Quality assessment

Methodological quality of included reviews was assessed independently by two reviewers (A.S.J.Y. and B.B.) using the ‘A MeaSurement Tool to Assess Systematic Reviews 2’ (AMSTAR-2) questionnaire (Shea et al., 2017). Any discrepancies were discussed among the reviewers. Reviews were considered to have used a comprehensive literature search strategy (item 4) if the authors included both trial registry search and citation search. Reviews were considered to have assessed the potential impact of risk of bias on the results of meta-analyses (item 12) if sensitivity or regression analyses were performed by excluding studies assessed as being of low quality. Reviews were considered to have accounted for risk of bias in individual studies when interpreting the results of the review (item 13) if certainty of evidence was graded using a validated tool. The overall quality of the review was rated as high, moderate, low or critically low based on the seven critical domains recommended by the AMSTAR-2 checklist items 2, 4, 7, 9, 11, 13 and 15. We did not exclude poor-quality systematic reviews from the overview of reviews. However, the results of poor-quality systematic reviews were interpreted in light of the specific quality considerations.

### Certainty assessment

The certainty of evidence of each outcome was extracted from the reviews if it was reported.

## Results

### Study selection

**Figure 1.**
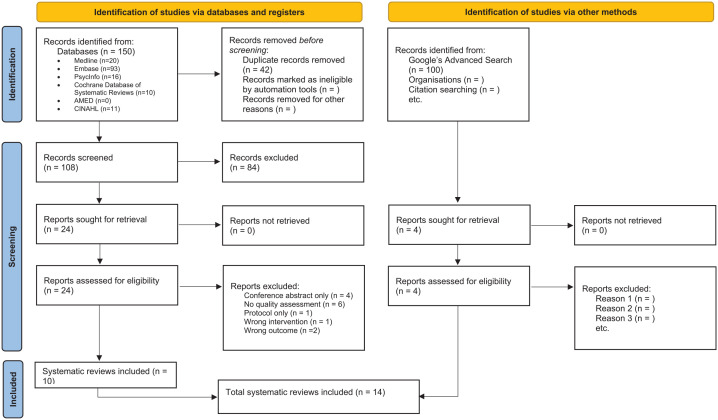
Reporting guideline for overviews of reviews of healthcare interventions: the Preferred Reporting Items for Overviews of Reviews (PRIOR) statement. Source: Gates et al. (2022).

A total of 150 records were identified from the 6 bibliometric databases (Figure 1). After removing duplicates, 108 titles and abstracts were screened, resulting in 24 full texts that were assessed for eligibility for inclusion. Fourteen full texts were excluded due to reasons such as wrong intervention, wrong outcome, no full article or no quality assessment (refer to Supplementary Table S2 for the list of excluded studies). In total, 14 systematic reviews were included in this overview, including 4 systematic reviews identified from grey literature.

### Study characteristics

Eleven reviews included meta-analyses, with only 3 exceptions (Breeksema et al., 2022; Heath et al., 2022; Varker et al., 2021). Ten reviews reported on both the safety and efficacy of MDMA-AP, while four reviews focused solely on either safety (Breeksema et al., 2022; Colcott et al., 2024) or efficacy (Hoskins et al., 2021; Luoma et al., 2020) (Table 1; refer to Table 2 for the general characteristics of each included review). All the reviews on efficacy were for PTSD, whereas the reviews on safety evaluated the adverse events reported in various psychiatric conditions, including PTSD, anxiety and alcohol use disorder. Ten systematic reviews included randomised or quasi-randomised trials only, while four also included observational studies (Breeksema et al., 2022; Mackey et al., 2022; Tedesco et al., 2021; Varker et al., 2021). The most recent literature search was up until 5 April 2024. The 14 reviews included 20 primary studies (including 5 unpublished trials) published between 2008 and 2023 and involved up to 353 participants. The 20 primary studies were conducted in the United States (*n* = 10), Canada (*n* = 4), Israel (*n* = 4), Switzerland (*n* = 2), Brazil (*n* = 1), Spain (*n* = 1), the United Kingdom (*n* = 1) and New Zealand (*n* = 1) (Supplementary Table S3). Two reviews included only patients with treatment-resistant PTSD (Illingworth et al., 2021; Tedesco et al., 2021). However, the criteria used to determine treatment resistance were not explicitly defined in the reviews. All reviews used one to three sessions of 50–125 mg MDMA (some with supplemental dosage, which was half of the initial dose) with psychotherapy as interventions (Table 3). The control group was either active placebo (25–40 mg of MDMA) or inactive placebo along with psychotherapy. The reviews used Cochrane Risk of Bias tool or NHMRC’s modified quality and risk of bias checklist to assess the risk of bias of RCTs. The two reviews focusing on safety also used Critical Appraisal Skills Programme (CASP), CONSORT Harms 2022 or qualitative assessment of methods to assess the quality of side effects-reporting. Four reviews assessed the certainty of evidence using the GRADE approach (Bahji et al., 2023; Colcott et al., 2024; Kisely et al., 2023; Varker et al., 2021), while the others used Institute for Clinical and Economic Review (ICER) Evidence Rating Matrix or Agency for Healthcare Research and Quality (AHRQ) Methods Guide for Comparative Effectiveness Reviews (Mackey et al., 2022; Mustafa et al., 2024). Mapping of primary studies showed a very high degree of overlap (CCA 28%) among the reviews (Supplementary Table S3). The majority of the primary studies were funded by the Multidisciplinary Association for Psychedelic Studies (MAPS). MAPS is a non-profit research and educational organisation that owns Lykos Therapeutics, the first company to file a new drug submission of MDMA-AP to the US FDA (Fierce Biotech, 2023).

**Table 1.** Summary of major outcomes reported in systematic reviews.

Outcomes	Summary of findings
Study characteristics	• Reported on both the safety and efficacy of MDMA-AP: *n* = 10 • Reported on safety only: *n* = 2 (Breeksema et al., 2022; Colcott et al., 2024) • Reported on efficacy only: *n* = 2 (Hoskins et al., 2021; Luoma et al., 2020)
	**Intervention group**: 50–125 mg MDMA-AP (1–3 sessions)
	**Comparator group**: active placebo (25–40 mg of MDMA) or inactive placebo along with psychotherapy
	**Risk of bias assessment tools**: Cochrane Risk of Bias tool, modified quality and risk of bias checklist (NHMRC, 1999), Critical Appraisal Skills Programme (CASP), CONSORT Harms 2022, or qualitative assessment
	**Certainty of evidence assessment**: GRADE approach, ICER Evidence Rating Matrix, AHRQ Methods Guide for Comparative Effectiveness Reviews
Methodology quality (assessed by authors using AMSTAR-2)	high = 4, moderate = 1, low = 3, critically low = 6
Efficacy of MDMA-AP	**PTSD symptoms**: SMD in CAPS score of −0.8 to −1.3 (Bahji et al., 2020, 2023; Green et al., 2023; Hoskins et al., 2021; Kisely et al., 2023; Mackey et al., 2022; Mustafa et al., 2024; Tedesco et al., 2021)
	**Response rates**: RR of 1.3 to 3.5 (compared to comparator group) (Bahji et al., 2020, 2023; Kisely et al., 2023; Mustafa et al., 2024; Tedesco et al., 2021)
	**Remission rates**: RR of 2.3 to 2.9 (compared to comparator group) (Bahji et al., 2020, 2023; Mustafa et al., 2024; Tedesco et al., 2021)
	**Loss of diagnosis**: RR of 1.70 (compared to comparator group) (Mustafa et al., 2024)
	**Depressive symptoms**: MD in Beck’s Depression Inventory of −10.8 to −11.1 (Green et al., 2023; Illingworth et al., 2021)
	**Daily functioning**: MD in Sheehan Disability Scale of −1.5 (Mustafa et al., 2024); SMD of −0.4 to −0.8 (Green et al., 2023; Mustafa et al., 2024)
	**Long-term outcomes** (change in PTSD symptoms after 2–74 months follow-up): SMD of −0.8 to −1.1 (Bahji et al., 2020; Tedesco et al., 2021)
Safety of MDMA-AP	**Any adverse events (immediate)**: OR of 1.7 to 3.5 (compared to comparator group) (Colcott et al., 2024)^a^
	**Any adverse effects (up to 7 days)**: OR of 1.6 (compared to comparator group) (Colcott et al., 2024)^a^
	**Adverse events of special interest**: No significant difference found
	**Rate of discontinuation**: No significant difference (Colcott et al., 2024) or lower risk of discontinuation in the intervention group: RR of 0.32 (Mustafa et al., 2024)
	**Long-term adverse events**: 2–4% of adverse events reported after 12 months (Colcott et al., 2024)

AHRQ, Agency for Healthcare Research and Quality; ICER, Institute for Clinical and Economic Review; MD, mean difference; MDMA-AP, MDMA-assisted psychotherapy; OR, odds ratio; SMD, standardised mean difference; RR, relative risk.

The statistical summaries reported for efficacy and safety outcomes are presented at a high level and should be interpreted within the context of each individual review and its certainty of evidence.

a Refer to individual review for incidence rate or RR of each adverse event.

**Table 2.** General characteristics of included reviews.

Author, year	Date of search	Study design	No. of studies (No. of participants)	PICO	Quality assessment tool	Certainty of evidence assessment tool	Remarks
Bahji et al. (2020)	December 2018	Randomised, quasi-randomised trials	5 (106)	P- patients with PTSD I- MDMA + psychotherapy C- pharmacotherapy, placebo, or non-pharmacotherapy (supportive care) O- clinically significant response/remission	Cochrane RoB tool	NA	• Included unpublished work and conference proceedings
Bahji et al. (2023)	15 May 2023	RCT	6 (182)	P- patients with depression, anxiety or PTSD I- MDMA alone or combined with therapy C- placebo/ active comparator O- symptom improvement and remission using validated psychometric instruments	Cochrane RoB tool	GRADE (did not grade each outcome)	NA
Breeksema et al. (2022)	28 July 2021	Quantitative and qualitative clinical studies	16 (266)	P- patients with a mental disorder I- classic serotonergic psychedelic or entactogenic drug C- NA O- adverse reactions	CASP (for qualitative studies); qualitative assessment of methods used to monitor or report adverse events, study inclusion and exclusion criteria, and the percentage of participants with prior experience with the drug	NA	• Studies reported adverse events either via spontaneous reporting (11 studies), systematic assessment (2 studies), or using UKU scale of secondary effects (1 study)
Colcott et al. (2024)	30 October 2023	Phase 2 and 3 clinical studies	13 (333)	P- patients with psychiatric conditions I- MDMA with psychotherapy C- NA O- side effects, study withdrawal	Cochrane RoB2 tool, CONSORT Harms 2022	GRADE	• Included population with various psychiatric conditions: PTSD (10 studies), autistic adults with social anxiety (1 study) various treatment-resistant disorders (1 study), alcohol use disorder (1 study) • No included RCT had adequate adherence to the CONSORT Harms 2022 recommendations (>70%), with median adherence rate of 50% (range 21–64%) • Comparison of adverse events published on ClinicalTrial.gov showed that 1487 non-serious adverse events (vs 661 in published articles) and 13 serious adverse events (vs 9 in published articles) were recorded
Green et al. (2023) ^a^	8 July 2022	RCTs	6 (169)	P- adults with PTSD I- MDMA-assisted psychotherapy C- active/ inactive placebo + psychotherapy O- clinically administered PTSD scale	Cochrane RoB tool	NA	• Reported analysis of secondary outcome measures
Heath et al. (2022)	2010 to 3 June 2022	RCT	8 (201)	P- population with PTSD I- MDMA ± other interventions (e.g. psychotherapy or psychological support) C- active or inactive comparator O- efficacy & safety	Domain-based approach adapted from Cochrane ROB tool, Jadad scoring system	NA	• Included studies published in other non-journal formats (e.g. from a clinical trial registry site or poster) when there was sufficient detail reported. • For crossover designs with persistent treatment effects, only reported on comparisons that preceded the crossover
Hoskins et al. (2021)	May 2018	RCT	4 (85)	P- patients with PTSD (at least 3 months) I- pharmacological-assisted psychotherapy C- psychotherapy, medication or psychotherapy + placebo O- symptom severity	Cochrane RoB tool	NA	• Other than MDMA-AP, review also assessed the use of other pharmacological approach, such as SSRI, SNRI, d-cycloserine, propranolol, yohimbine, cortisol, methylene blue • Commented that MDMA-AP used a non-evidence-based psychotherapy model, which has not been evaluated against other trauma-focused psychological therapies • All four studies were funded and managed by a single sponsor (MAPS)
Illingworth et al. (2021)	February 2020	RCT	4 (85)	P- patients with treatment-resistant PTSD I- MDMA + psychotherapy C- active/ inactive placebo + psychotherapy O- CAPS-4 score (after at least 3 weeks) & BDI score, adverse effects immediately or at 7 day follow-up (secondary)	Cochrane RoB tool	NA	NA
Kisely et al. (2023)	August 2021	RCT	6 (182)	P- humans (excluding healthy volunteers) I- MDMA + psychotherapy C- active/inactive placebo + psychotherapy O- psychiatric outcomes (after at least 2 weeks), response & remission (secondary)	Cochrane RoB tool	GRADE	• Excluded pre-prints that had not been peer-reviewed • Only used results of the first phase/arm of treatment in crossover trials
Luoma et al. (2020)	March 2019	RCT	4 (81)	P- patients with diagnosed psychiatric condition I- MDMA, psilocybin, ayahuasca, *N,N*-dimethyltryptamine (DMT), or LSD + therapy C- placebo O- efficacy	Cochrane RoB tool	NA	• For studies that used cross-over designs, only data collected before the cross-over was included. • Tested for moderating variables (gender, race, age, number of dosing sessions, number of psychotherapy sessions, sample size) using meta-regressions
Mackey et al. (2022)	26 April 2022	RCT, cohort studies, pre-post studies, case series	12 (353)	P- Adults with mental health and/or substance use disorders I- psychedelics C- any (e.g. placebo, treatment as usual) O- efficacy, harms	Cochrane RoB tool	AHRQ Methods Guide for Comparative Effectiveness Reviews	• Included data from clinical trial registry that are not peer-reviewed
Mustafa et al. (2024)	5 April 2024	RCT	2 (194)	(from protocol) P- adults with a diagnosis of moderate-to-severe PTSD I- MDMA-assisted Therapy (Lykos-specific psychotherapy) C- Lykos-specific non-assisted psychotherapy O- efficacy, harms	Cochrane RoB tool	ICER Evidence Rating Matrix	• Excluded articles indexed as guidelines, letters, editorials, narrative reviews, case reports or news items. • Review was supplemented with data from conference proceedings, regulatory documents, information submitted by manufacturers, and other grey literature when the evidence met ICER standards
Tedesco et al. (2021)	24 March 2021	Randomised, quasi-randomised and uncontrolled trials	10 (168)	P- patients with treatment-resistant PTSD I- MDMA + psychotherapy C- pharmacotherapy, placebo, and psychotherapy O- clinically significant response/remission	Cochrane RoB tool, ROBINS-I	NA	• Included unpublished work • All 10 studies in meta-analyses were funded by MAPS
Varker et al. (2021)	18 October 2019	RCT and observational studies	4 (86)	P- adults with PTSD I- MDMA alone or with another treatment C- active/inactive treatment, placebo or wait-list alternatives O- changes in PTSD symptoms and/or diagnostic status	Modified quality and risk of bias checklist (NHMRC, 1999)	GRADE	NA

AHRQ, Agency for Healthcare Research and Quality; BDI, Beck Depression Inventory; CAPS, Clinician Administered PTSD Scale; CASP, Critical Appraisal Skills Programme; GRADE, Grading of Recommendations Assessment, Development, and Evaluation; ICER, Institute for Clinical and Economic Review; NA, not available; PICO, population, intervention, comparator, outcome; RoB, risk of bias; UKU, Udvalg for Kliniske Undersøgelser/Scandinavian Society for Psychopharmacology.

a Non-peer-reviewed pre-print.

### Methodology quality

Four reviews were assessed as being of high methodological quality (Colcott et al., 2024; Heath et al., 2022; Kisely et al., 2023; Mustafa et al., 2024) (Supplementary Table S4). For the critical domains, most of the reviews did not account for risk of bias in individual studies when interpreting the results of the review (7/14), did not report the prior establishment of a review protocol (5/14) and did not provide a list of excluded studies with the reasons for exclusion (3/14). For the non-critical domains, the most common gaps were not reporting the sources of funding (5/14) and not assessing the potential impact of risk of bias on the results (5/14).

For the review that assessed the quality of adverse event reporting based on the CONSORT Harms 2022 checklist, it was reported that none of the primary studies included achieved more than 70% adherence to the checklist, with median adherence rate of 50% (Colcott et al., 2024). Colcott et al. (2024) also compared the adverse events reporting rates between the published articles and their registers on ClinicalTrial.gov and found that 56% of non-serious and 31% of serious adverse events on the clinical trial register were not reported in the published articles.

### Efficacy of MDMA-assisted psychotherapy

Efficacy outcomes were measured as improvement in PTSD symptoms measured via Clinician Administered PTSD Scale (CAPS) score, response rate or remission rate. Some reviews also performed meta-analyses on other outcomes, such as depressive symptoms and functionality (Illingworth et al., 2021; Mustafa et al., 2024).

#### Effect on PTSD symptom scores

Overall, the reviews reported a large reduction in PTSD symptoms, with standardised mean difference (SMD) ranging from 0.8 to 1.3 (Table 1; refer to Table 3 for the detailed results of each review). There was low certainty of evidence that 100–125 mg of MDMA-AP was significantly associated with improvement in PTSD outcomes at 4–12 weeks compared to active placebo (SMD: −0.86; 95% confidence interval [CI]: [−1.23, −0.50]) (Kisely et al., 2023). Mustafa et al. (2024) included Phase 3 clinical trials only and reported that 80–120 mg of MDMA-AP reduced CAPS-5 score by mean difference (MD) of −10.18 (95% CI: [−13.80, −6.56]) (SMD: −0.8; 95% CI: [−0.49, −1.1]) compared to inactive placebo after 18 weeks, but the overall net health benefit was graded as ‘insufficient’ evidence. Mackey et al. (2022) compared MDMA-AP to both active and inactive placebo and reported low certainty evidence of reduction in PTSD symptoms (overall SMD: −0.91; 95% CI: [−1.33, −0.50]; against active placebo: −0.98; 95% CI: [−1.92, −0.05]; against inactive placebo: −0.88; 95% CI: [−1.77, 0.02]). Other reviews did not stratify the outcomes according to different doses and control groups and reported a large overall improvement in PTSD symptom scores, with SMD ranging from 0.93 to 1.30 (Bahji et al., 2020, 2023; Green et al., 2023; Hoskins et al., 2021; Tedesco et al., 2021).

**Table 3.** Summary of key results of included reviews.

Author, year	Population	MDMA dosage for intervention and control	Findings on efficacy (Certainty of evidence)	Findings on safety (Certainty of evidence)	Methodological quality (AMSTAR-2)
Bahji et al. (2020)	No summary of population	I: 50–125 mg (± supplemental half dose) (1–3 sessions) C: 25–40 mg or inactive placebo	**PTSD symptoms scores** SMD: −1.30 [−0.66, −1.94] **Response rate** RR: 3.47 [1.70, 7.06] **Remission rate** RR (*k* = 5): 2.63 [1.37, 5.02] **Long-term outcomes (after 2–74 months)** SMD: 1.10 [0.42, 1.78]	4/5 studies did not report any MDMA-related serious adverse events. 1/5 study reported MDMA-related increased depressive symptoms and suicidal ideation	Low
Bahji et al. (2023)	No summary of population	I: 50–180 mg (no mention of supplemental dose) (1–3 sessions which are 1–5 weeks apart) C: 25–40 mg or inactive placebo	**PTSD symptoms scores** SMD (*k* = 6): −0.95 [−1.28, −0.62] **Response rate** RR (*k* = 6): 3.21 [1.78, 5.79] **Remission rate** RR (*k* = 6): 2.32 [1.53, 3.53] **Long-term outcomes** One study reported outcomes at 17 and 74 months for 16 participants. Results showed sustained symptomatic relief, with two participants experiencing a relapse. (Overall evidence graded as low to very low but did not grade each outcome independently.)	(no MDMA-specific data) For overall psychedelic-assisted therapy, no significant association found with retention in treatment (RR: 1.00 [0.96–1.04]), the overall number of dropouts (RR: 0.88 [0.56–1.37]), or dropouts due to adverse events (RR: 1.50 [0.38–5.89]). (Overall evidence graded as low to very low but did not grade each outcome independently.)	Moderate
Breeksema et al. (2022)	No summary of population	I: 75–125 mg (± supplemental half dose) (1–3 sessions) C: 25–40 mg or inactive placebo	**Not applicable**	Acute AE: jaw clenching and/or tight muscles, headaches, nausea, fatigue, lack of appetite, anxiety (occurred more often in the MDMA than in the placebo groups, with some exceptions). Only one study reported the severity of the acute AEs (Oehen et al., 2013). Late AE: fatigue, lack of appetite, low mood, insomnia, need for more sleep, increased irritability, headache, difficulty concentrating, and anxiety (uncertain whether it was more common in control or intervention group). Some AE lasted up to 2 months after the final session	Critically low
Colcott et al. (2024)	No summary of population	I: 50–125 mg + psychotherapy (± supplemental half dose) (1–3 sessions) C: 25–40 mg or inactive placebo. Some RCTs were cross-over studies which used same dosage as intervention group for open-label arms	**Not applicable**	**Adverse effects (immediate)** Phase 2 studies: OR of any AE: 1.67 [1.12, 2.49] [recalculated RR: 1.39] (very low^g^) (statistically significant for anxiety and jaw-clenching) TEAE: not statistically significant (very low^g^) Phase 3 studies (*n* = 194): OR of any TEAE: 3.51 [2.76, 4.46] [recalculated RR: 3.11] (moderate^h^) (statistically significant for muscle tightness, decreased appetite, nausea, excessive perspiration, feeling cold, restlessness, dilated pupils, jaw clenching/tight jaw, uncontrolled eye movements, feeling jittery, non-cardiac chest pain/discomfort, blurred vision and chills) Adverse event of special interest (suicidality and cardiac): not statistically significant (low^i^) **Adverse effects (up to 7 days)** Phase 2 studies: OR of any AE: 1.59 [1.12, 2.24] (very low^g^) **Withdrawal** No difference in the odds of withdrawal across all Phase 2 and 3 studies. All reasons for withdrawal were not statistically significant between 2 groups (moderate^h^) **Long-term adverse effects** Pooled analysis on six Phase 2 trials (*n* = 107) reported that participants experienced worsened mood (4%), increased nightmares or intrusive memories (2%), difficulty feeling emotions (2%), avoiding people or places (2%), increased anxiety (2%), excessive vigilance (2%) up to 12 months after the trial (effects not separated based on intervention or control arm; Jerome et al., 2020)	High
Green et al. (2023) ^a^	No summary of population	I: 120–125 mg (2–3 sessions) (no mention of supplemental dose) C: 25–40 mg or inactive placebo	**CAPS score** SMD = 0.93 [0.60 to 1.25] **Dissociation severity** (Disassociation Experience scale) MD: −9.7 [−13.39 to −6.12] **Daily** **Functioning** (Global Assessment of Functioning scale, Sheehan Disability Scale) SMD: 0.82 [0.10 to 1.73] **Depression** (Beck’s Depression Inventory) MD: −11.13 [−19.35 to −2.92] **Sleep disturbance severity** (Pittsburgh Sleep Quality Index): not statistically significant	**Heart rate** MD: 12.88 [0.97 to 24.79]	Low
Heath et al. (2022)	• Phase 2 trials (*n* = 105): mean age = 40.5 years, 58.5% female. 87.6% White, mean duration of PTSD symptoms = 18 years, nearly all participants had tried at least one psychotherapy modality prior to study (only two participants had not) • Phase 3 trials (*n* = 90): All participants were chronic (⩾6 months) and severe (CAPS-5 total score ⩾35), mean age = 41 years, 66% female, 77% White, 90% non-Hispanic/Latino, mean duration of PTSD symptoms = 14 years, 21% had dissociative PTSD subtype, 2.2% did not have a history of psychotherapy	I: 75–125 mg (± supplemental half dose) (2–3 sessions) C: 25–40 mg MDMA (± supplemental half dose) or inactive placebo	**CAPS score** • Large effect sizes in reducing CAPS scores in the Phase 3 trial (Cohen’s *d* = 0.9 with three treatment sessions) • Five out of seven Phase 2 trials are suggestive for the efficacy of MDMA-assisted therapy over comparator **Loss of diagnosis (no longer met DSM-5 diagnostic criteria)** 67% vs 32% (intervention vs control group) **Re mission** 33% vs 5% (intervention vs control group)	**Serious AE** During the blinded study period: suicidality, breast cancer, lower limb fracture, ruptured ovarian cyst, central nervous system metastasis, clavicle, syncope During an unknown follow-up period: extrasystole exacerbation, events of suicidal ideation, depression, appendicitis **Psychiatric AEs** (attributed to MDMA during a Phase 3 trial) bruxism, restlessness, intrusive thoughts, nervousness, stress, insomnia **Non-psychiatric TEAEs** • Most frequently (⩾15%) reported in the MDMA compared to control group included mydriasis, muscle tightness, nausea, decreased appetite, feeling cold and hyperhidrosis • An increased incidence (⩾5% higher than placebo) of blurred vision, increased blood pressure and postural dizziness occurred in the active MDMA group, and investigators considered these events to be related to MDMA • No reports of events related to MDMA misuse/ abuse	High
Hoskins et al. (2021)	No summary of population	I: 75–125 mg (no mention of supplemental dose) (total 12 sessions including preparatory and integration sessions) C: 25–40 mg or inactive placebo	**PTSD symptoms scores** SMD: −1.09 [−1.60, −0.58]	NR	Critically low
Illingworth et al. (2021)	No summary of population	I: 75–125 mg (no mention of supplemental dose) C: Active or inactive placebo	**CAPS-4 scores** 75 mg vs active placebo- MD: −46.90 [−58.78, −35.02] 100 mg vs active placebo- statistically insignificant 125 mg vs active placebo- MD: −20.98 [−34.35, −7.61] 125 mg vs inactive placebo- MD: −33.20 [−40.53, −25.87] **BDI** 75 mg vs active placebo- MD: −10.80 [−20.39, −1.21]. **Long-term outcomes not reported**	**Higher incidence of jaw-clenching in intervention group** 125 mg vs inactive placebo- RR: 4.22 [1.49, 11.95] 75 mg vs active placebo- POR: 13.46 [1.44, 125.80] 125 mg (per patient) vs active placebo- POR: 7.34 [1.98, 27.71] 125 mg (per session) vs active placebo- POR: 4.08 [1.04, 15.99] 150 mg vs active placebo- RR: 8.67 [1.21, 61.91] There were some statistically significant differences for ‘Low mood in session’, ‘Nausea in session’, ‘Lack of appetite within 7 days of session’ were reported in one dosage, but they did not follow a dose–response relationship, suggesting non-causative aetiology	Critically low
Kisely et al. (2023)	• Where it was recorded, participants were overwhelmingly White/European. • Trials generally excluded people with a personal or family history of psychosis, personal history of mania, repeated violence towards others and a recent personal history of a suicide attempt, as well as those with current drug or alcohol use disorders	I: 50–187.5 mg (including supplemental dose) (1–3 sessions) C: 25–40 mg MDMA or inactive placebo	**PTSD symptoms after 4–12** **weeks** 100–125 mg vs active placebo- SMD: −0.86 [−1.23, −0.50] (low^b^) 125–187.5 mg vs active placebo- SMD: −1.21 [−2.20, −0.22] (very low^c^) **Response (30% reduction in CAPS score) after 4–8 weeks** Not statistically significant **Remission (no longer meet diagnostic criteria)** Not statistically significant **Long-term outcomes not reported**	**Adverse effects (immediate)** Higher incidence of jaw-clenching in intervention group- RR: 2.35 [1.15, 4.80] Higher incidence of anxiety and reduced appetite but statistically insignificant **Adverse effects (up to 7 days)** Higher incidence of fatigue, jaw clenching and reduced appetite, but all statistically insignificant (certainty of evidence not rated for safety outcomes)	High
Luoma et al. (2020)	Race was reported in seven studies (85% reported being White) and gender was reported in all studies (53% identified as women)	I: 75–125 mg (± supplemental half dose) (2–3 sessions) C: 25–40 mg or inactive placebo	**PTSD symptoms scores** effect size = Hedges *g* = 1.22 No significant difference in the overall effect size between MDMA and other classic psychedelics	NR	Critically low
Mackey et al. (2022)	All studies included participants with moderate (total severity score 40–59 on CAPS-4 and 23–34 on CAPS-5) or severe (⩾60 on CAPS-4 and ⩾35 on CAPS-5) symptoms at baseline. Most studies included participants who had been experiencing PTSD symptoms for years and had previously undergone at least one medication or psychotherapy trial	I: 80–125 mg (± supplemental half dose) (1–3 sessions) C: 25–40 mg MDMA (± supplemental half dose) or inactive placebo	**CAPS score** SMD: −0.91 [−1.33, −0.50] (overall) SMD: −0.98 [−1.92, −0.05] (low-dose placebo) SMD: −0.88 [−1.77, 0.02] (inactive placebo) (low^e^)	Only one study reported a serious adverse event attributed to MDMA, a participant with premature ventricular contractions (PVCs) at baseline experienced an increase in PVCs requiring an overnight hospital stay for observation. Other transient side effects of MDMA include symptoms related to mood (e.g. anxiety, irritability, restlessness), sleep (e.g. fatigue, insomnia), sensation (e.g. dizziness, impaired balance), pain (e.g. headache, muscle tension), and gastrointestinal systems (e.g. nausea, vomiting, low appetite)	Low
Mustafa et al. (2024)	• Diagnosis of PTSD for approximately 15 years at study baseline, mean age = 40 years old, 69% were female, 71% were White, ~40% had previous lifetime experience with MDMA	I: 80–120 mg (± supplemental half dose) (three sessions) C: Inactive placebo	**PTSD symptoms after 18** **weeks** MD: −10.18 [−13.80, −6.56] SMD: −0.8 [−0.49 to −1.1] **Response** (⩾10-point reduction in CAPS-5) RR: 1.32 [1.11, 1.58] **Loss of diagnosis** (⩾10-point reduction in CAPS-5 and not meeting PTSD diagnostic criteria) RR: 1.70 [1.26, 2.29] **Remission rate** (loss of diagnosis and a total CAPS-5 score of ⩽11) RR: 2.86 [1.58, 5.16] **Sheehan Disability Scale** (for functional impairment) MD: −1.48 [−1.60; −1.36] SMD: 0.42 [0.17; 0.66] **Long-term outcomes** 2–3 sessions of 75–125 mg of MDMA-AP compared to control group (0–40 mg of MDMA-AP) change in CAPS-4 score mean = −44.8 (SE: 2.82, *n* = 100) (baseline to treatment exit (1–2 months after final MDMA session) mean = -50.6 (SE: 2.68, *n* = 91) (baseline to LTFU (12 months after final MDMA session) mean = −5.2 (SE: 2.29) (treatment exit to LTFU) loss of diagnosis 67% (LTFU) vs 56% (treatment exit) (Overall certainty of evidence is rated as ‘insufficient^d^’ after considering the net health benefit using the ICER Evidence Rating Matrix.)	**Adverse effects (immediate)** • TEAEs were common, occurring in 96–100% of participants • Higher risk in MDMA-AP group compared to control (muscle tightness, decreased appetite, bruxism, hyperhidrosis [excessive sweating], fatigue, restlessness and insomnia) but RR not calculated • These events were generally of short duration and characterised as mild to moderate in terms of severity • Increases in blood pressure, body temperature and heart rate were observed, but were transient and expected **Rate of discontinuation** • Lower risk in intervention group (RR: 0.32; 95% CI: [0.12, 0.85]) • Authors cautioned that the difference in dropout may be partially explained by the functional unblinding seen in both MAPP trials and the corresponding heightened expectancy effect **Adverse event of special interest** • Reports of cardiac AEs, such as palpitations and tachycardia, but they were infrequent and mild in severity • No reported data on long-term cardiovascular events • No events of MDMA abuse reported during or after the therapy • Very low certainty that there is no increased risk of suicidal ideation with MDMA-AP (RR: 0.89; 95% CI: [0.64, 1.24]) (Overall certainty of evidence is rated as ‘insufficient^d^’ after considering the net health benefit using the ICER Evidence Rating Matrix.)	High
Tedesco et al. (2021)	Average age was 40.6 years old, 78.2% of participants had comorbid depression, 38.2% had comorbid anxiety	I: 50–187.5 mg (± supplemental half dose) (1–3 sessions) C: 25–40 mg or inactive placebo or none	**PTSD symptoms scores** SMD: −0.93 [−0.51, −1.36] **Response rate** RR: 3.1 [1.29, 7.45] **Remission rate** RR (*k* = 8): 2.96 [1.63, 5.39] Long-term outcome **MDMA improves PTSD symptoms scores after 1–74 months** SMD: −0.81 [−0.40, −1.23]	Adverse events reported in one study (diminished appetite, anxiety, headache, jaw tightness, tinnitus, nausea, asthenia, fatigue, acute sinusitis, nasopharyngitis, upper respiratory tract infection, disturbance in attention, tremor, tics, dysuria and erythema) all resolved by the 6-month follow-up period One possible drug-related serious event of depression with suicidal ideation was reported	Critically low
Varker et al. (2021)	No summary of population	I: 75–125 mg (± supplemental half dose) (2–3 sessions) C: 25–40 mg or inactive placebo	Improvements in clinician-rated or self-reported PTSD symptoms and self-reported physical responses to stress were significantly greater in the MDMA group compared to the placebo group. Long-term data for the MDMA group collected 17 to 74 months after the original study’s final MDMA session showed that on average improvements were maintained following the intervention (Overall evidence graded as moderate^f^ did not grade each outcome independently.)	The side effects reported included moderate insomnia, loss of appetite, restlessness, tight jaw, thirst, feeling cold, dizziness, headaches and impaired gait/balance (Mithoefer et al., 2011, 2018; Oehen et al., 2013; Ot’alora G et al., 2018). There was one serious adverse event that was deemed to have been possibly related to a drug treatment, with the participant developing an acute increase in ventricular contractions during an open-label session (Mithoefer et al. 2018). Overall safety data has indicated a favourable risk-to-benefit ratio for moderate doses of pure MDMA for treating those with PTSD (Mithoefer et al., 2011, 2013; Oehen et al., 2013). (Overall evidence graded as moderate^f^ but did not grade each outcome independently.)	Critically low

AE, adverse effects; LTFU, Long-term follow-up; POR, peto odds ratio; k, number of primary studies; TEAE, treatment emergent adverse event.

a Non-peer-reviewed pre-print; ^b^Rated down for indirectness and publication bias; ^c^Rated down for indirectness, imprecision and publication bias; ^d^Rated down due to concern in risk of bias (functional unblinding of participants, reporting bias of investigators/therapists, safety data were not collected by independent and blinded raters), publication bias (some predefined outcomes were not consistently reported), indirectness (generalisability concerns to a population naive to psychedelics), suitability of CAP-5 to measure outcome, uncertainty around long-term follow-up data, use of an unproven therapy as the comparison arm, insufficient data on the abuse potential of MDMA; ^e^Rated down due to study limitations and imprecision; ^f^Noted some limitations in quality but rated all four domains (treatment assignment, control of selection bias, blinding, outcome assessment) as low risk of bias; ^g^Rated down due to risk of bias, indirectness and imprecision; ^h^Rated down due to risk of bias; ^i^Rated down due to risk of bias and imprecision.

Bahji et al. (2020); Bahji et al. (2023); Breeksema et al. (2022); Colcott et al. (2024); Green et al. (2023); Heath et al. (2022); Hoskins et al. (2021); Illingworth et al. (2021); Jerome et al. (2020); Kisely et al. (2023); Luoma et al. (2020); Mackey et al., (2022); Mustafa et al. (2024); Tedesco et al. (2021); Varker et al. (2021).

#### Effect on response rates

The relative risk (RR) of response rate reported in the reviews ranged from 1.3 to 3.5 (Table 1). Different reviews defined response rates differently. Mustafa et al. (2024) defined a ‘response’ as a more than 10-point reduction in CAPS-5 score and reported an RR of 1.32 (95% CI: [1.11, 1.58]) based on two Phase 3 RCTs, but the evidence was considered insufficient. Kisely et al. (2023) defined a clinically significant response as a more than 30% reduction in CAPS-4 score. It reported an RR of 3.33 (95% CI: [0.98, 11.37]) in the intervention group after 4–8 weeks, but the finding was from a single study and considered very low certainty. Bahji et al. (2023) did not explicitly state the criteria used to define a clinically significant response. It conducted a meta-analysis on six primary studies with varying definitions of a ‘response’ and reported an RR of 3.21 (95% CI: [1.78, 5.79]) among participants given MDMA-AP compared to the control group. Similarly, Tedesco et al. (2021) included eight RCTs and reported an RR of 3.1 (95% CI: [1.29, 7.45]) among those undergoing MDMA-AP. Notably, two of the RCTs included in the meta-analysis were not peer-reviewed and no sensitivity analysis was conducted to explore the possible impact.

#### Effect on loss of diagnosis or remission rates

The reviews reported remission rates of 2.3 to 2.9 (RR compared to comparator groups) (Table 1). Remission was generally defined as no longer meeting the diagnostic criteria for PTSD based on CAPS assessment. Mustafa et al. (2024) further differentiated between ‘loss of diagnosis’ and ‘remission’, where ‘remission’ was defined as loss of diagnosis with an additional condition of total CAPS-5 score of ⩽11. It reported a higher rate of loss of diagnosis (RR: 1.70; 95% CI: [1.26, 2.29]) and remission (RR: 2.86; 95% CI: [1.58, 5.16]) among those receiving MDMA-AP compared to inactive placebo. Heath et al. (2022) reported a higher proportion of those in the intervention group than control group achieved loss of diagnosis (67% vs 32%) and remission (33% vs 5%) at the primary endpoint. Tedesco et al. (2021) reported a remission rate of up to three times higher in the intervention group (RR: 2.96; 95% CI: [1.63, 5.39]) based on eight primary studies, whereas Bahji et al. (2023) reported an RR of 2.32 (95% CI: [1.53, 3.53]) based on six RCTs.

#### Effect on other outcomes

Two reviews reported that MDMA-AP was associated with a significant mean reduction in depressive symptoms as measured by the Beck Depression Inventory scores, with MD of −11.13 (95% CI: [−19.35, −2.92]) (Green et al., 2023) and −10.8 (95% CI: [−20.39, −1.21]) (compared to active placebo) (Illingworth et al., 2021). Mustafa et al. (2024) reported that 80–120 mg of MDMA-AP had a moderate effect (SMD: 0.42; 95% CI: [0.17, 0.66]) on reducing functional impairment, as measured by a mean reduction of −1.48 (95% CI: [−1.60, −1.36]) on the Sheehan Disability Scale.

#### Long-term outcomes

Long-term follow-up studies of six Phase 2 trials (*n* = 100) of up to 12 months after the final MDMA administration (except one trial with a 74-month follow-up due to delay in follow-up initiation) reported a sustained reduction in CAPS-4 score (mean = −44.8, SE: 2.82) and an increase in percentage of participants with loss of diagnosis from treatment exit to long-term follow-up (67% vs 56%) (Mustafa et al., 2024). However, the review cautioned against the potential confounding effects from receiving other forms of PTSD treatment or medication during long-term follow-up. The analysis that informed the long-term follow-up findings was later retracted due to protocol violations amounting to unethical conduct by trial researchers at one of the study sites (Jerome et al., 2024). Two reviews reported a large effect size of MDMA-AP on PTSD symptoms improvement with SMD of 0.8–1.1 after 2–74 months of follow-up, but the interpretation was unclear due to the mixing of participants from the intervention and control groups during cross-over (Bahji et al., 2020; Tedesco et al., 2021).

### Safety of MDMA-assisted psychotherapy

Colcott et al. (2024) reported that 11/13 of the included studies relied on spontaneous adverse event reporting instead of using systematic checklists. The other systematic reviews did not distinguish between passive versus systematic adverse event assessment. The adverse events were reported as immediate adverse events which arose during therapy sessions, adverse events that persisted within 7 days following administration and long-term adverse events.

#### Immediate adverse events

Colcott et al. (2024) separated its meta-analyses of immediate adverse events by their clinical trial phases because Phase 2 and 3 studies used different methods to report adverse events. The included Phase 2 studies categorised adverse events into ‘spontaneously reported actions’ (defined as expected adverse events based on findings from healthy volunteer studies) and ‘treatment emergent adverse events (TEAE)’ (defined as events not on the expected reaction list or which continued for more than 7 days after administration session). Phase 3 studies did not make any distinctions between the two categories but monitored additionally for Adverse Events of Special Interest (AESI) relating to cardiac function, suicide risk and MDMA abuse, misuse or diversion, as advised by the US FDA. The review reported very low certainty evidence of an increased odds of experiencing *any* adverse events during medication sessions (odds ratio [OR]: 1.67; 95% CI: [1.12, 2.49]; recalculated RR: 1.39) among participants receiving MDMA-AP compared to control group, with significant higher odds of anxiety (OR: 4.84; 95% CI: [1.38, 16.97]; recalculated RR: 1.88) and jaw clenching (OR: 4.72; 95% CI: [1.01, 22]; recalculated RR: 3.28]) (Colcott et al., 2024). Kisely et al. (2023) also included Phase 2 clinical studies only in its meta-analyses, but it did not pool the findings of all adverse events to calculate the RR of experiencing *any* adverse events. Similarly, it reported significantly higher risk of jaw-clenching (RR: 2.35; 95% CI: [1.15, 4.80]) in the MDMA-AP group compared to control group, but the certainty of evidence was not graded (Kisely et al., 2023).

For Phase 3 studies (*n* = 194 participants), TEAEs were reported by 96–100% of participants, but they were transient with mild to moderate severity (Mustafa et al., 2024). There was moderate-quality evidence indicating that MDMA-AP was associated with increased odds (OR = 3.51; 95% CI: [2.76, 4.46]; recalculated RR: 3.11) of *any* adverse events (including significant risks for muscle tightness, decreased appetite, nausea, excessive perspiration, feeling cold, restlessness, dilated pupils, jaw clenching, uncontrolled eye movements, feeling jittery, non-cardiac chest pain, blurred vision and chills) during the treatment period compared to psychotherapy only (Colcott et al., 2024).

#### Adverse events up to 7 days after administration

Within 7 days following medication, the meta-analysis showed that the risk of experiencing any adverse events was higher in the intervention than control group (OR: 1.59; 95% CI: [1.12, 2.24]; recalculated RR: 1.34) (Colcott et al., 2024).

#### Adverse events of special interest

No significant difference was found in the AESI risk between the intervention and control groups, possibly due to the small number of events (Colcott et al., 2024). There were reports of cardiac adverse events, such as palpitations and tachycardia, but they were infrequent and mild in severity (Mustafa et al., 2024). Long-term data on cardiovascular events was not available. No event of MDMA abuse was reported during or after the therapy for the Phase 3 trials (Mustafa et al., 2024). There was no statistical difference between the intervention and control groups regarding the risk of suicidal ideation (RR: 0.89; 95% CI: [0.64, 1.24]) (Mustafa et al., 2024).

#### Rate of discontinuation

Mustafa et al. (2024) reported a lower risk of discontinuation in the intervention group (RR: 0.32; 95% CI: [0.12, 0.85]) compared to control group among Phase 3 clinical trials, while Colcott et al. (2024) did not report any difference in the odds of withdrawal across all Phase 2 and 3 studies. However, the authors cautioned that the lower dropout rate may be due to the functional unblinding that might have heightened the participant’s expectancy (Mustafa et al., 2024).

#### Long-term adverse events

Jerome et al. (2020) conducted a pooled analysis of six Phase 2 trials (*n* = 107) and reported that participants experienced worsened mood (4%), increased nightmares or intrusive memories (2%), difficulty feeling emotions (2%), avoiding people or places (2%), increased anxiety (2%) and excessive vigilance (2%) up to 12 months after the trial (Colcott et al., 2024). However, the study did not separate the adverse events in the intervention and control arms due to the open-label cross-over trial design and the publication was retracted on 10 August 2024 due to protocol violations and failure in disclosing affiliation with the funder of the trials (Jerome et al., 2024).

### Certainty of evidence

Three reviews rated the certainty of evidence down due to serious concern in the ‘indirectness’ domain as the findings might not be generalisable to the general population due to the stringent recruitment criteria and the high percentage of participants (up to 40%) with history of MDMA use (Colcott et al., 2024; Kisely et al., 2023; Mustafa et al., 2024). Two reviews reduced the certainty of evidence due to the risk of bias in primary studies, which was caused by functional unblinding of participants, reporting bias of participants and investigators motivated (or pressured) by the community’s strong belief in the potential of psychedelics, and collection of safety data not done by independent and blinded assessors (Colcott et al., 2024; Mustafa et al., 2024). Three reviews found that the findings were affected by imprecision, likely due to the small sample size (Colcott et al., 2024; Kisely et al., 2023; Mackey et al., 2022). Concerns for publication bias were also raised in two reviews as some predefined outcomes were not consistently reported (Kisely et al., 2023; Mustafa et al., 2024).

## Discussion

Four of 14 systematic reviews included in the overview were assessed as being high quality. Based on the four high-quality systematic reviews, there was insufficient or low certainty evidence that 80–125 mg of MDMA-AP results in a reduction in PTSD scores when compared to psychotherapy with active or inactive placebo. There was very low certainty evidence that MDMA-AP improved response and remission rate. Long-term follow-up data were available in Phase 2 trials for up to 12 months, but the evidence was potentially confounded by other PTSD treatments or medications. There was moderate certainty evidence that MDMA-AP was associated with an increased risk of experiencing any adverse events during the medication session, including muscle tightness, decreased appetite, nausea, excessive perspiration, feeling cold, restlessness, dilated pupils, jaw clenching, uncontrolled eye movements, feeling jittery, non-cardiac chest pain discomfort, blurred vision and chills. MDMA-AP may increase the risk of adverse events up to 7 days, but the evidence was of very low certainty. The reviews reported no significant difference in the risk of AESI relating to cardiac function or suicidality in Phase 3 trials, possibly due to the limited sample size.

There are gaps in how systematic reviews have reported psychotherapy, supplemental dosage and duration of interventions. Eleven of 14 systematic reviews did not compare and contrast the psychotherapy sessions that accompanied the MDMA administrations, except 3 reviews (Bahji et al., 2023; Heath et al., 2022; Mackey et al., 2022). Variations were observed in the number of integration sessions, which ranged from 2 to 4 sessions after each administration session (Mackey et al., 2022), and the qualification and training of therapist, where the Phase 3 trials specifically required at least one of its therapists to have a Master’s degree and the therapists had to go through a 9-day and 67-hour training programme (Heath et al., 2022). The lack of reporting in systematic reviews is probably contributed by the lack of reporting of the psychological intervention or psychotherapy in psychedelic clinical trials, where more than half of the 33 psychedelic clinical trials in a systematic review failed to indicate the use of a therapy manual (52%) or provide the reference of the manual (64%), and 42% did not report the therapist’s credentials (Brennan et al., 2023). Since psychological treatments are proven to contribute to improvement in PTSD symptoms (Lee et al., 2016; Watts et al., 2013; Weber et al., 2021), the lack of reporting or standardisation of psychotherapy in MDMA-AP might pose uncertainty on the disentanglement of the effect of psychological intervention from the efficacy of MDMA and limit the translation of MDMA-AP from clinical trials to clinical practice. The psychotherapies provided in those trials were not evidence-based treatments for PTSD, and there is no evidence of their effectiveness for PTSD as stand-alone treatments (Halvorsen et al., 2021). In addition, the reporting of supplemental dosage within and across systematic reviews was inconsistent and not standardised. Most reviews did not account for the supplemental dosage and the percentage of participants who received it when reporting the dosage, making it difficult to summarise the final dosage used in evidence generation. The duration of intervention for some pooled analyses was unclear or not reported in some reviews, limiting the interpretation of data in terms of effect durability. Future systematic reviews should ensure comprehensive investigation of the psychotherapy component, supplemental dosage, and duration of intervention to improve comparability of data across reviews.

Systematic reviews defined ‘treatment response’ and ‘treatment resistant’ differently. Two reviews focused on people with treatment-resistant PTSD (Illingworth et al., 2021; Tedesco et al., 2021), but there was inconsistency in the definition of treatment resistant. Tedesco et al. (2021) defined it as those with a longer than 6-month diagnosis and have received prior pharmaceutical and psychotherapy treatment, whereas Illingworth et al. (2021) accepted definitions assigned by the authors of the primary studies. Similarly, when analysing the response rate of MDMA-AP, some reviews combined primary studies with different definitions of a ‘clinically significant response’ for the meta-analyses. In fact, the challenge of operational definitions of treatment response, nonresponse or resistant is not unique to MDMA-AP; the field of PTSD treatment has always faced challenges in standardising these outcomes (Forbes et al., 2019; Howes et al., 2022; Sippel et al., 2018). There has been efforts to propose models to define treatment resistant, including The Emory Treatment Resistance Interview for PTSD (E-TRIP) (Dunlop et al., 2014) and the staged model of treatment-resistant PTSD (Sippel et al., 2018), but there is yet to be an agreed clinical decision algorithm for treatment-resistant PTSD (Forbes et al., 2019; Howes et al., 2022). Similarly, there are wide variations in terms of the criteria clinical trials used to define ‘treatment response’, including a minimum score reduction, a minimum percentage reduction, a cutoff score derived from a predefined statistical formula or a predefined cutoff score (Varker et al., 2020). This variability in operational definitions highlights the need for standardised criteria to ensure reproducibility of outcomes in follow-up research and to improve the applicability of findings to the clinical setting.

The findings on efficacy demonstrate substantial benefits of MDMA-AP in improving PTSD symptoms, loss of diagnosis and remission compared to psychotherapy alone. The improvement on PTSD symptoms with effect size of SMD 0.8–1.3 reported in the reviews are comparable to the efficacy of combined somatic or cognitive therapies (SMD −1.69; 95% CI: [−2.66, −0.73]) and trauma-focused cognitive behavioural therapy (SMD −1.46; 95% CI: [−1.87, −1.05]), which used waitlist as control (Mavranezouli et al., 2020). However, the evidence is of low to very low certainty due to the high risk of bias, indirectness, and imprecision in most systematic reviews. All reviews reported concerns regarding unblinding among participants and investigators after MDMA administration, which contributed to risk of bias in outcome assessment or expectancy bias. The challenge in blinding is not unique to MDMA and is commonly raised in other drugs with psychoactive properties, such as ketamine, benzodiazepines, gabapentinoids, opiates and stimulants (Butler et al., 2022). For reviews that reported the summary characteristics of the population (Heath et al., 2022; Kisely et al., 2023; Luoma et al., 2020; Mackey et al., 2022; Mustafa et al., 2024; Tedesco et al., 2021), there were concerns of the generalisability of the findings to clinical practice due to the limited representation of participants, who are predominantly White (71–88%), female (53–69%), had comorbid depression (78–91%) and with previous lifetime experience with MDMA (~40%). Those with psychotic disorders, personality disorders, active substance use disorders, high suicide risk and high-risk cardiovascular conditions were generally excluded from trials (Heath et al., 2022; Mustafa et al., 2024). Since the current evidence is mainly from the United States, there are also potential issues concerning the generalisability of the findings to clinical practice in other health systems and settings in different countries. Most reviews also cautioned the interpretation of the findings due to the limited sample size (more so after stratification of intervention according to MDMA dosage and types of placebo in meta-analyses), which affected the precision of outcomes. Future studies with larger sample size and a diverse population are essential to ascertain the efficacy of MDMA-AP. In addition, reviews should address how unblinding might affect the interpretation of results.

The certainty of safety data in the systematic reviews varies from moderate to very low. Colcott et al. (2024) reported that reliance on spontaneous reporting of adverse events rather than application of systematic checklists may have resulted in adverse events being underestimated. Four reviews noted discrepancies between adverse events reported in published articles and trial registries (Breeksema et al., 2022; Colcott et al., 2024; Heath et al., 2022; Mustafa et al., 2024), with up to 31% of serious adverse events registered on ClinicalTrials.gov not being reported in the corresponding published articles (Colcott et al., 2024). This overview also identified significant gaps in long-term safety data as the included reviews only provided follow-up data of up to 12 months. Adverse events for cardiovascular events, abuse and misuse potential, and suicidal risk were mainly collected in Phase 3 trials with long-term follow-up data not yet available (Mustafa et al., 2024). These findings align with the recent US FDA meeting to review evidence from Lykos Therapeutics for MDMA registration, which highlighted the lack of data on hepatotoxicity, hyponatremia, drug–drug interactions and abuse-related adverse events (US Food and Drug Administration, 2024). On the other hand, Breeksema et al. (2022) posited that adverse events in psychedelic studies need to be redefined because certain adverse events are ‘challenging but potentially therapeutically beneficial’. For example, categorising ‘anxiety’ as adverse events might prevent participants from revisiting difficult events or thoughts, which might be a necessary therapeutic process. These considerations highlight the need for future studies to employ systematic checklists for adverse events assessment and adhere to rigorous reporting practices. With the recent reclassification of psychedelics in Australia, the lack of long-term data also offers a unique opportunity for real-world data collection and monitoring locally since the current evidence are mainly from the United States, Canada, Switzerland and Israel.

There are currently no MDMA products listed in the Australian Register of Therapeutic Goods. However, the Therapeutic Goods Administration (TGA) rescheduled MDMA to allow certain patients to access MDMA-AP with specific safeguards (Therapeutic Goods Administration, 2023a, 2023b). These safeguards include requiring prescribing psychiatrists to be authorised under the Authorised Prescriber scheme and adherence to treatment protocols approved by Human Research Ethics Committees and TGA. Before making possible recommendations on the use of MDMA-AP in clinical practice, a comprehensive range of factors need to be considered. A multidisciplinary team has been engaged to develop a clinical practice guideline on this topic. The Guideline Development Group will use the GRADE evidence-to-decision framework to consider factors including evidence of benefits and harms, certainty of the evidence, patient values and preferences, resources, equity, acceptability and feasibility (Alonso-Coello et al., 2016, 2017). The high-quality systematic reviews identified in this overview will be used to assist in generating evidence profiles that will be considered when making recommendations.

## Strengths and limitations

The strength of this overview lies in its comprehensive search strategy, which included both bibliometric databases and grey literature. It includes not only peer-reviewed journal articles but also reports from academic institutions, government agencies and non-profit organisation. The overview critically summarises and evaluates the methodologies used in various systematic reviews and meta-analyses.

However, this overview also reflects the inherent limitations of the included reviews, including small sample size, high risk of bias, selected population and low certainty of evidence. The heterogeneity between studies regarding design of psychotherapy sessions, MDMA dosage, supplemental dosage, types of placebo and population necessitates cautious interpretation of the findings in line with the study designs. The study design heterogeneity and low certainty of evidence reported by the reviews also limit the applicability of the findings to clinical practice.

## Conclusion

In conclusion, there are four high-quality systematic reviews that summarised the efficacy and safety of MDMA-AP. The most common methodological gap related to systematic reviews not accounting for risk of bias in individual studies when interpreting efficacy and safety data. Systematic reviews reported on the effects of MDMA-AP in improving PTSD symptoms, response and remission rate, but did not critically evaluate the psychotherapy session, supplemental dosage, and intervention duration. The certainty of evidence for efficacy was rated as low or very low in the systematic reviews due to challenges in blinding, limited generalisability, and small sample sizes in clinical trials. In terms of safety, one systematic review comprehensively compared the risk of various adverse events between the intervention and control groups. However, this review concluded that the evidence about adverse events was limited by weaknesses in adverse event assessment and reporting, and the relatively short follow-up periods. Future systematic reviews should address these issues to ensure evidence from clinical trials can be used to inform clinical practice regarding the use of MDMA-AP for PTSD.

## Supplemental Material

sj-docx-1-anp-10.1177_00048674251315642 – Supplemental material for Safety and efficacy of methylenedioxymethamphetamine (MDMA)-assisted psychotherapy in post-traumatic stress disorder: An overview of systematic reviews and meta-analyses Supplemental material, sj-docx-1-anp-10.1177_00048674251315642 for Safety and efficacy of methylenedioxymethamphetamine (MDMA)-assisted psychotherapy in post-traumatic stress disorder: An overview of systematic reviews and meta-analyses by Alene Sze Jing Yong, Suzie Bratuskins, Musa Samir Sultani, Brooke Blakeley, Christopher G Davey and J Simon Bell in Australian & New Zealand Journal of Psychiatry
