# Supplementary material for: Safety and efficacy of methylenedioxymethamphetamine (MDMA)-assisted psychotherapy in post-traumatic stress disorder: An overview of systematic reviews and meta-analyses
Source: Aust N Z J Psychiatry. 2025 Feb 20;59(4):339–60. doi: 10.1177/00048674251315642 (PMC11924292; doi:10.1177/00048674251315642)
Supplement: sj-docx-1-anp-10.1177_00048674251315642 – Supplemental material for Safety and efficacy of methylenedioxymethamphetamine (MDMA)-assisted psychotherapy in post-traumatic stress disorder: An overview of systematic reviews and meta-analyses [file sj-docx-1-anp-10.1177_00048674251315642.docx]

Supplementary Tables S1. Search strategy for each database.

| Ovid MEDLINE(R) and Epub Ahead of Print, In-Process, In-Data-Review & Other Non-Indexed Citations, Daily and Versions <1946 to May 09, 2024> | | |
| --- | --- | --- |
| 1 | N-Methyl-3,4-methylenedioxyamphetamine.mp. or N-Methyl-3,4-methylenedioxyamphetamine/ | 4358 |
| 2 | (MDMA or midomafetamine or ecstasy or methamphetamine or methylenedioxyamphetamine or methylamphetamine).mp. [mp=title, book title, abstract, original title, name of substance word, subject heading word, floating sub-heading word, keyword heading word, organism supplementary concept word, protocol supplementary concept word, rare disease supplementary concept word, unique identifier, synonyms, population supplementary concept word, anatomy supplementary concept word] | 22777 |
| 3 | (MDMA assisted therapy or MDMA assisted psychotherapy or psychedelic assisted therapy or psychedelic assisted psychotherapy or substance assisted therapy or substance assisted psychotherapy).mp. [mp=title, book title, abstract, original title, name of substance word, subject heading word, floating sub-heading word, keyword heading word, organism supplementary concept word, protocol supplementary concept word, rare disease supplementary concept word, unique identifier, synonyms, population supplementary concept word, anatomy supplementary concept word] | 362 |
| 4 | 1 or 2 or 3 | 22967 |
| 5 | exp Stress Disorders, Post-Traumatic/ | 43004 |
| 6 | (ptsd or post traumatic stress disorder).mp. [mp=title, book title, abstract, original title, name of substance word, subject heading word, floating sub-heading word, keyword heading word, organism supplementary concept word, protocol supplementary concept word, rare disease supplementary concept word, unique identifier, synonyms, population supplementary concept word, anatomy supplementary concept word] | 41919 |
| 7 | 5 or 6 | 57735 |
| 8 | (systematic review or meta analys*s).mp. [mp=title, book title, abstract, original title, name of substance word, subject heading word, floating sub-heading word, keyword heading word, organism supplementary concept word, protocol supplementary concept word, rare disease supplementary concept word, unique identifier, synonyms, population supplementary concept word, anatomy supplementary concept word] | 500858 |
| 9 | 4 and 7 and 8 | 20 |

| Embase Classic+Embase <1947 to 2024 May 09> | |  |
| --- | --- | --- |
| 1 | exp midomafetamine/ | 3087 |
| 2 | (N-Methyl-3,4-methylenedioxyamphetamine or MDMA or ecstasy or methamphetamine or methylenedioxyamphetamine or methylamphetamine).mp. [mp=title, abstract, heading word, drug trade name, original title, device manufacturer, drug manufacturer, device trade name, keyword heading word, floating subheading word, candidate term word] | 37371 |
| 3 | (MDMA assisted therapy or MDMA assisted psychotherapy or psychedelic assisted therapy or psychedelic assisted psychotherapy or substance assisted therapy or substance assisted psychotherapy).mp. [mp=title, abstract, heading word, drug trade name, original title, device manufacturer, drug manufacturer, device trade name, keyword heading word, floating subheading word, candidate term word] | 410 |
| 4 | 1 or 2 or 3 | 38238 |
| 5 | exp posttraumatic stress disorder/ | 85065 |
| 6 | (ptsd or stress disorder or post traumatic stress disorder).mp. [mp=title, abstract, heading word, drug trade name, original title, device manufacturer, drug manufacturer, device trade name, keyword heading word, floating subheading word, candidate term word] | 94421 |
| 7 | 5 or 6 | 94708 |
| 8 | (systematic review or meta analys*s).mp. [mp=title, abstract, heading word, drug trade name, original title, device manufacturer, drug manufacturer, device trade name, keyword heading word, floating subheading word, candidate term word] | 774444 |
| 9 | 4 and 7 and 8 | 93 |

| APA PsycInfo <1806 to May Week 2 2024> | |  |
| --- | --- | --- |
| 1 | exp Methylenedioxymethamphetamine/ | 2330 |
| 2 | (N-Methyl-3,4-methylenedioxyamphetamine or MDMA or midomafetamine or ecstasy or methamphetamine or methylenedioxyamphetamine or methylamphetamine).mp. [mp=title, abstract, heading word, table of contents, key concepts, original title, tests & measures, mesh word] | 10989 |
| 3 | (MDMA assisted therapy or MDMA assisted psychotherapy or psychedelic assisted therapy or psychedelic assisted psychotherapy or substance assisted therapy or substance assisted psychotherapy).mp. [mp=title, abstract, heading word, table of contents, key concepts, original title, tests & measures, mesh word] | 379 |
| 4 | 1 or 2 or 3 | 11267 |
| 5 | exp Posttraumatic Stress Disorder/ | 42375 |
| 6 | (ptsd or stress disorder or post traumatic stress disorder).mp. [mp=title, abstract, heading word, table of contents, key concepts, original title, tests & measures, mesh word] | 61685 |
| 7 | 5 or 6 | 61687 |
| 8 | (systematic review or meta analys*s).mp. [mp=title, abstract, heading word, table of contents, key concepts, original title, tests & measures, mesh word] | 86254 |
| 9 | 4 and 7 and 8 | 16 |

| EBM Reviews - Cochrane Database of Systematic Reviews <2005 to May 8, 2024> | |  |
| --- | --- | --- |
| 1 | (N-Methyl-3,4-methylenedioxyamphetamine or MDMA or midomafetamine or ecstasy or methamphetamine or methylenedioxyamphetamine or methylenedioxymethamphetamine or methylamphetamine).mp. [mp=title, short title, abstract, full text, keywords, caption text] | 62 |
| 2 | (MDMA assisted therapy or MDMA assisted psychotherapy or psychedelic assisted therapy or psychedelic assisted psychotherapy or substance assisted therapy or substance assisted psychotherapy).mp. [mp=title, short title, abstract, full text, keywords, caption text] | 2 |
| 3 | 1 or 2 | 62 |
| 4 | (Posttraumatic Stress Disorder or post traumatic stress disorder or ptsd or stress disorder).mp. [mp=title, short title, abstract, full text, keywords, caption text] | 252 |
| 5 | (systematic review or meta analys*s).mp. [mp=title, short title, abstract, full text, keywords, caption text] | 10167 |
| 6 | 3 and 4 and 5 | 10 |

| AMED (Allied and Complementary Medicine) <1985 to March 2024> | |  |
| --- | --- | --- |
| 1 | (N-Methyl-3,4-methylenedioxyamphetamine or MDMA or midomafetamine or ecstasy or methamphetamine or methylenedioxyamphetamine or methylenedioxymethamphetamine or methylamphetamine).mp. [mp=abstract, heading words, title] | 27 |
| 2 | (MDMA assisted therapy or MDMA assisted psychotherapy or psychedelic assisted therapy or psychedelic assisted psychotherapy or substance assisted therapy or substance assisted psychotherapy).mp. [mp=abstract, heading words, title] | 0 |
| 3 | exp Stress disorders post traumatic/ | 842 |
| 4 | (Posttraumatic Stress Disorder or post traumatic stress disorder or ptsd or stress disorder).mp. [mp=abstract, heading words, title] | 852 |
| 5 | (systematic review or meta analys*s).mp. [mp=abstract, heading words, title] | 7641 |
| 6 | 1 or 2 | 27 |
| 7 | 3 or 4 | 1097 |
| 8 | 5 and 6 and 7 | 0 |

| CINAHL <inception to May 2024> | |  |
| --- | --- | --- |
| 1 | (MH "Methamphetamine+") OR "methamphetamine" | 5,809 |
| 2 | MDMA or midomafetamine or ecstasy or N-Methyl-3,4-methylenedioxyamphetamine or methylenedioxyamphetamine or methylamphetamine | 1,881 |
| 3 | MDMA assisted therapy or MDMA assisted psychotherapy or psychedelic assisted therapy or psychedelic assisted psychotherapy or substance assisted therapy or substance assisted psychotherapy | 159 |
| 4 | S1 OR S2 OR S3 | 6,693 |
| 5 | (MH "Stress Disorders, Post-Traumatic+") OR "post traumatic stress disorder" | 29,822 |
| 6 | ptsd or stress disorder | 37,287 |
| 7 | S5 OR S6 | 37,379 |
| 8 | systematic review or meta analysis | 255,030 |
| 9 | S4 AND S7 AND S8 | 11 |

Supplementary S2. List of excluded articles.

Conference abstract only (n = 4)

1. Bright S and Williams M (2022) Mdma-Assisted Psychotherapy. *Australian and New Zealand Journal of Psychiatry* 56(SUPPL 1): 45.
2. Kisely S, Connor M and Somogyi A (2022) Do Psilocybin or Methylenedioxyme Thamphetamine (Mdma) Improve Mental, Behavioural or Developmental Disorders Either as Stand-Alone Treatments or in Combination with Psychotherapy? *Australian and New Zealand Journal of Psychiatry* 56(SUPPL 1): 179.
3. Lunsky I, Gutierrez G, Bahji A, et al. (2021) Psychedelics for the treatment of mental disorders: A systematic review and network meta-analysis. *Neuropsychopharmacology* Conference: 60th Annual Meeting of the American College of Neuropsychopharmacology, ACNP 2021. San Juan Puerto Rico. 46: 279.
4. Sa Couto J, Da Luz B, Rodrigues J, et al. (2022) Methylenedioxymethamphetamine-assisted Psychotherapy For Posttraumatic Stress Disorder: A Review. *European Psychiatry* 65(Supplement 1): S672-S673.

No quality assessment (n = 6)

1. Amoroso T and Workman M (2016) Treating posttraumatic stress disorder with MDMA-assisted psychotherapy: A preliminary meta-analysis and comparison to prolonged exposure therapy. *Journal of Psychopharmacology* 30(7): 595-600.
2. Reiff CM, Richman EE, Nemeroff CB, et al. (2020) Psychedelics and Psychedelic-Assisted Psychotherapy. *American Journal of Psychiatry* 177(5): 391-410.
3. Smith KW, Sicignano DJ, Hernandez AV, et al. (2022) MDMA-Assisted Psychotherapy for Treatment of Posttraumatic Stress Disorder: A Systematic Review With Meta-Analysis. *Journal of Clinical Pharmacology* 62(4): 463-471.
4. Wheeler SW and Dyer NL (2020) A systematic review of psychedelic-assisted psychotherapy for mental health: An evaluation of the current wave of research and suggestions for the future. *Psychology of Consciousness: Theory, Research, and Practice* 7(3): 279-315.
5. White CM (2014) 3,4-Methylenedioxymethamphetamine's (MDMA's) Impact on Posttraumatic Stress Disorder. *Annals of Pharmacotherapy* 48(7): 908-915.
6. Yao Y, Guo D, Lu TS, et al. (2024) Efficacy and safety of psychedelics for the treatment of mental disorders: A systematic review and meta-analysis. *Psychiatry Research* 335: 115886.

Protocol only (n = 1)

1. Zuljevic MF, Vidak M, Vukojevic J, et al. (2022) 3,4-methylenedioxymethamphetamine (MDMA)-assisted psychotherapy for post-traumatic stress disorder (PTSD) in adults. *Cochrane Database of Systematic Reviews* 2022(7) (no pagination).

Wrong intervention (n = 1)

1. Bouchet L, Sager Z, Yrondi A, et al. (2024) Older adults in psychedelic-assisted therapy trials: A systematic review. *Journal of Psychopharmacology* 38(1): 33-48.

Wrong outcome (n =2)

1. Ching TH, Williams MT, Wang JB, et al. (2022) MDMA-assisted therapy for posttraumatic stress disorder: A pooled analysis of ethnoracial differences in efficacy and safety from two Phase 2 open-label lead-in trials and a Phase 3 randomized, blinded placebo-controlled trial. *Journal of Psychopharmacology* 36(8): 974-986.
2. Morgan L (2020) MDMA-assisted psychotherapy for people diagnosed with treatment-resistant PTSD: what it is and what it isn't. *Annals of General Psychiatry* 19(1): 1-7.

Supplementary Table S3. Mapping of primary studies in systematic review.

| Study (location) | Kisely 2023 | Hoskins 2021 | Bahji 2020 | Bahji 2023 | Illingworth 2021 | Tedesco 2021 | Luoma 2020 | Varker 2021 | Breeksema 2022 | Mustafa 2024 | Heath 2022 | Mackey 2022^a^ | Green 2023 | Colcott 2024^b^ | **Total** |
| --- | --- | --- | --- | --- | --- | --- | --- | --- | --- | --- | --- | --- | --- | --- | --- |
| Bouso 2008 (Spain) | yes | no | yes | yes | no | yes | no | no | yes | no | no | yes | no | yes | **7** |
| Danforth 2018 (USA) | no | no | no | no | no | no | no | no | yes | no | no | no | no | yes | **2** |
| Jardim 2021 (Brazil) | no | no | no | no | no | no | no | no | yes | no | no | yes | no | yes | **3** |
| Mitchell 2021 (USA, Canada, Israel) | yes | no | no | yes | no | no | no | no | yes | yes | yes | yes | yes | yes | **8** |
| Mitchell 2023 (USA, Israel) | no | no | no | no | no | no | no | no | no | yes | no | no | no | yes | **2** |
| Mithoefer 2011 (USA) | yes | yes | yes | yes | yes | yes | yes | yes | yes | no | yes | yes | yes | yes | **13** |
| Mithoefer 2018 (USA) | yes | yes | yes | yes | yes | yes | yes | yes | yes | no | yes | yes | yes | yes | **13** |
| Monson 2020 (USA) | no | no | no | no | no | yes | no | no | yes | no | no | yes | no | yes | **4** |
| MP-16/ NCT03282123^c^ (USA) | no | no | no | no | no | yes | no | no | no | no | no | no | no | no | **1** |
| MP-17/ NCT03485287^c^ (Canada) | no | no | no | no | no | yes | no | no | no | no | no | no | no | no | **1** |
| MP-4/ NCT01958593^c^ (Canada) | no | no | no | no | no | yes | no | no | no | no | yes | yes | no | no | **3** |
| MP-9/ NCT01689740^c^ (Israel) | no | no | no | no | no | yes | no | no | no | no | yes | yes | yes | no | **4** |
| NCT00402298^c,d^ (Israel) | no | no | no | no | no | no | no | no | no | no | yes | no | no | no | **1** |
| Oehen & Gasser 2022 (Switzerland) | no | no | no | no | no | no | no | no | no | no | no | no | no | yes | **1** |
| Oehen 2013 (Switzerland) | yes | yes | yes | yes | yes | yes | yes | yes | yes | no | yes | yes | yes | yes | **13** |
| Ot'alora 2018 (USA) | yes | yes | yes | yes | yes | yes | yes | yes | yes | no | yes | yes | yes | yes | **13** |
| Searchfield 2020 (New Zealand) | no | no | no | no | no | no | no | no | yes | no | no | no | no | no | **1** |
| Sessa 2021 (UK) | no | no | no | no | no | no | no | no | yes | no | no | no | no | yes | **2** |
| Wang 2021 (USA, Canada) | no | no | no | no | no | no | no | no | no | no | no | yes | no | no | **1** |
| Wolfson 2020 (USA) | no | no | no | no | no | no | no | no | yes | no | no | no | no | no | **1** |
| **Total** | **6** | **4** | **5** | **6** | **4** | **10** | **4** | **4** | **12** | **2** | **8** | **11** | **6** | **12** | **94** |

^a^ Mithoefer 2019 was not listed as primary studies because it was a pooled analysis of other included primary studies; ^b^Jerome 2020 was not listed as primary studies because it was a pooled analysis of other included primary studies; ^c^ not peer-reviewed; ^d^terminated early

Supplementary Table S4. Methodological quality of included reviews assessed using AMSTAR 2.

|  | Kisely 2023 | Illingworth 2021 | Tedesco 2021 | Bahji 2020 | Bahji 2023 | Luoma 2020 | Hoskins 2021 | Varker 2021 | Breeksema 2022 | Colcott 2024 | Mustafa 2024 | Heath 2022 | Green 2023 | Mackey 2022 |
| --- | --- | --- | --- | --- | --- | --- | --- | --- | --- | --- | --- | --- | --- | --- |
| **Q1** | Yes | Yes | Yes | Yes | Yes | Yes | Yes | Yes | Yes | Yes | Yes | Yes | Yes | Yes |
| **Q2** | Yes | Yes | No | Yes | Yes | No | No | No | No | Yes | Yes | Yes | Yes | Yes |
| **Q3** | Yes | Yes | Yes | Yes | No | No | No | No | Yes | Yes | Yes | Yes | No | No |
| **Q4*** | Yes | Partial yes | Yes | Yes | Partial yes | Partial yes | Yes | Partial yes | Yes | Partial yes | Yes | Yes | Partial yes | Yes |
| **Q5** | Yes | Yes | Yes | Yes | Yes | Yes | Yes | Yes | Yes | Yes | Yes | Yes | Yes | Yes |
| **Q6^[[1]](#footnote-1)^** | Yes | Yes | Yes | Yes | Yes | Partial yes | Yes | Partial yes | Partial yes | Partial yes | Partial yes | Partial yes | Yes | Partial yes |
| **Q7** | Yes | No | No | Partial yes | Partial yes | Partial yes | No | Partial yes | Partial yes | Partial yes | Partial yes | Yes | Partial yes | Yes |
| **Q8** | Yes | No | Yes | Yes | Yes | Yes | Yes | Yes | Partial yes | Yes | Yes | Yes | Partial yes | Yes |
| **Q9*** | Yes | Partial yes | Yes | Yes | Yes | Yes | Yes | Yes | Yes | Yes | Yes | Yes | Yes | Yes |
| **Q10** | Yes | No | Yes | Yes | No | No | Yes | No | No | No | Yes | Yes | Yes | Yes |
| **Q11*** | Yes | Yes | Yes | Yes | Yes | Yes | Yes | NA | NA | Yes | Yes | NA | Yes | Yes |
| **Q12** | Yes | No | Yes | No | Yes | No | No | NA | NA | Yes | Yes | NA | Yes | No |
| **Q13*** | Yes | No | No | No | Yes | No | No | Yes | No | Yes | Yes | Yes | No | Yes |
| **Q14** | Yes | No | Yes | Yes | Yes | Yes | No | No | Yes | Yes | Yes | Yes | No | Yes |
| **Q15*** | Yes | No | Yes | Yes | Yes | Yes | No | NA | NA | Yes | Yes | NA | Yes | No |
| **Q16** | Yes | Yes | Yes | Yes | Yes | Yes | Yes | Yes | Yes | Yes | Yes | No | Yes | Yes |
| **Overall quality** | High | Critically low | Critically low | Low | Moderate | Critically low | Critically low | Critically low | Critically low | High | High | High | Low | Low |

*Critical domains

| Q1 | Did the research questions and inclusion criteria for review include the components of PICO |
| --- | --- |
| Q2 | Did the report of the review contain an explicit statement that the review methods were established prior to the conduct of the review and did the report justify any significant deviations from the protocol? |
| Q3 | Did the review authors explain their selection of the study designs for inclusion in the review? |
| Q4 | Did the review authors use a comprehensive literature search strategy |
| Q5 | Did the review authors perform study selection in duplicate |
| Q6 | Did the review authors perform data extraction in duplicate? |
| Q7 | Did the review authors provide a list of excluded studies and justify the exclusions? |
| Q8 | Did the review authors describe the included studies in adequate detail? |
| Q9 | Did the review authors use a satisfactory technique for assessing the risk of bias (RoB) in individual studies that were included in the review? |
| Q10 | Did the review authors report on the sources of funding for the studies included in the review? |
| Q11 | If meta-analysis was performed did the review authors use appropriate methods for statistical combination of results? |
| Q12 | If meta-analysis was performed, did the review authors assess the potential impact of RoB in individual studies on the results of the meta-analysis or other evidence synthesis? |
| Q13 | Did the review authors account for RoB in individual studies when interpreting/ discussing the results of the review? |
| Q14 | Did the review authors provide a satisfactory explanation for, and discussion of, any heterogeneity observed in the results of the review? |
| Q15 | If they performed quantitative synthesis did the review authors carry out an adequate investigation of publication bias (small study bias) and discuss its likely impact on the results of the review? |
| Q16 | Did the review authors report any potential sources of conflict of interest, including any funding they received for conducting the review? |

1. This domain is rated as ‘Partial yes’ if data extraction was not done in duplicate, but the extracted data was checked by another reviewer. [↑](#footnote-ref-1)
